# Celiac Axis Resection with Distal Pancreatectomy (Modified Appleby Procedure) Allows for R0 Resection of Pancreatic Body and Tail Mass Following Neoadjuvant Therapy: Case Report and Literature Review

**DOI:** 10.1089/crpc.2016.0011

**Published:** 2016-06-01

**Authors:** Mackenzie Morris, Thea Price, Zachary Callahan, Charles J. Yeo

**Affiliations:** ^1^ Sidney Kimmel Medical College, Thomas Jefferson University, Philadelphia, Pennsylvania.; ^2^ Department of Surgery, Thomas Jefferson University Hospital, Philadelphia, Pennsylvania.

**Keywords:** chemotherapy, distal pancreatectomy, celiac axis resection, pancreatic cancer

## Abstract

**Background:** The modified Appleby procedure has been developed for cancer of the pancreatic body or tail with celiac axis invasion, historically classified as unresectable disease. Post-Appleby resection, the source of arterial blood to the liver is the superior mesenteric artery, which supplies the gastroduodenal artery and ultimately feeds the proper hepatic artery. In cases of inadequate collateralization, preoperative coiling of the common hepatic artery (CHA) or intraoperative reconstruction via an aorto-hepatic bypass has been described.

**Method:** We describe a 74-year-old female with a pancreatic mass that was initially determined to be unresectable. She underwent extensive combination neoadjuvant chemotherapy. A favorable response was evidenced by a decrease in serum CA 19–9 levels. After 7 months, she was restaged and offered a distal pancreatectomy (DP) with the possibility of a modified Appleby procedure due to potential tumor involvement of the proximal CHA.

**Results:** Intraoperatively, tumor was identified along the CHA traveling proximally to the celiac axis. Therefore, a modified Appleby procedure with DP and splenectomy was performed without the need for reconstruction of the CHA. Postoperative specimen pathology showed residual pancreatic ductal adenocarcinoma with marked treatment effects. The pathology confirmed an R0 resection. The patient followed our postpancreatic surgery care pathway. She remains well 7 months postoperatively.

**Conclusion:** A pancreatic body or tail mass encasing the celiac vessels should not be an immediate referral for palliative care. Recent evidence shows that successful R0 resection can be achieved following neoadjuvant therapy. In fact, patients who have undergone a successful modified Appleby procedure show survival outcomes similar to patients with less advanced cancer who underwent standard DP. The modified Appleby procedure used in conjunction with neoadjuvant therapy can achieve complete resection in select patients previously thought to be unresectable.

## Introduction

Despite advances in adjuvant therapy and perioperative care, pancreatic adenocarcinoma continues to carry a grim prognosis. According to the American Cancer Society, 48,960 new cases of pancreatic cancer are expected to be diagnosed in the United States this year and 40,560 pancreatic cancer-related deaths are expected to occur. For all stages combined, the 1- and 5-year relative survival rates are 28% and 7%, respectively.^1^ Risk factors for pancreatic cancer include family history, smoking, long-standing diabetes mellitus, and chronic pancreatitis. People with a first-degree relative with pancreatic cancer are at an increased risk by at least a factor of 2. There is no effective screening tool and the onset of symptoms usually occurs late in the course of disease.

A daunting 90% of patients with a diagnosis of pancreatic cancer die from the disease. Surgical resection continues to be the mainstay of curative treatment, however, only 15–20% of patients at presentation are candidates for surgical resection. Pancreaticoduodenectomy (the Whipple procedure) is the classic procedure to resect tumors located in the head, neck, and uncinate of the pancreas. Tumors in the body and tail of the pancreas are commonly resected by a distal pancreatectomy (DP) with en bloc splenectomy.^2^

Patients with adenocarcinoma of the body and tail of the pancreas tend to present late in the disease course and involvement of the celiac axis or its proximal branches has historically been considered a contraindication to attempted resection. Dr. Appleby originally described a resection including the celiac axis in the 1950s as an approach to advanced gastric cancer.^3^ In the 1970s, the DP-celiac axis resection (DP-CAR) or modified Appleby procedure was adapted for pancreatic cancer with celiac axis invasion. Sufficient antegrade hepatopetal flow of arterial blood through the gastroduodenal artery (GDA) is needed for perfusion of the liver and stomach to avoid ischemic gastropathy and hepatic ischemia.^4^ A recent case series showed increased short-term survival in patients who underwent DP-CAR when compared to reports of patients with similar disease progression deemed unresectable. High morbidity was noted in resected patients, but few complications were serious and the majority of patients had resolution of their presenting symptom, intractable back pain.^5^

In recent years, an interest has developed in using neoadjuvant therapy as a way to increase resectability of locally advanced pancreatic cancer. Specifically, the four drug combination FOLFIRINOX has shown favorable conversion from unresectable disease to resectable disease.^6–8^

## Case Report

**FIG. 1.**
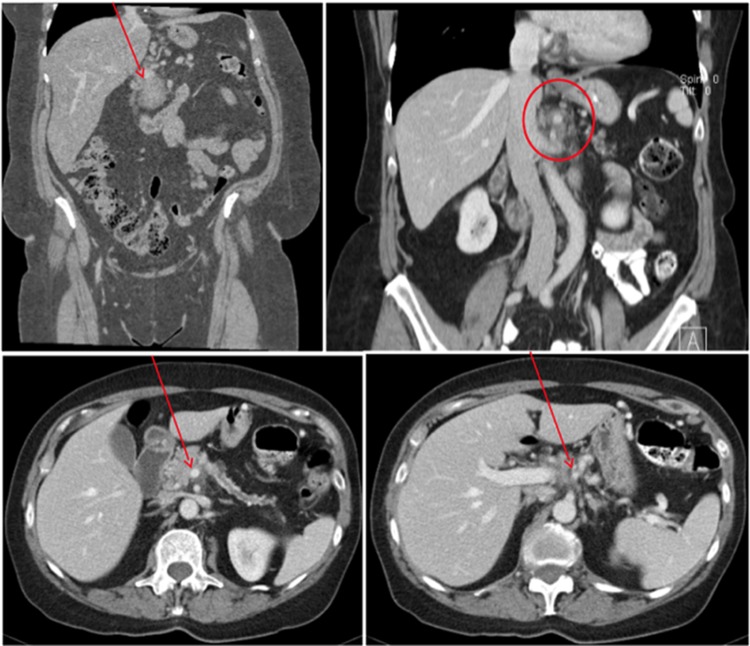
Initial CT scan at presentation showing a locally advanced tumor (arrow) of the proximal pancreatic body (upper left) and dilatation of the main pancreatic duct with involvement of the major visceral vessels, including the celiac artery (upper right), portal vein (lower left; arrow), and splenic artery and vein (lower right; arrow).

**FIG. 2.**
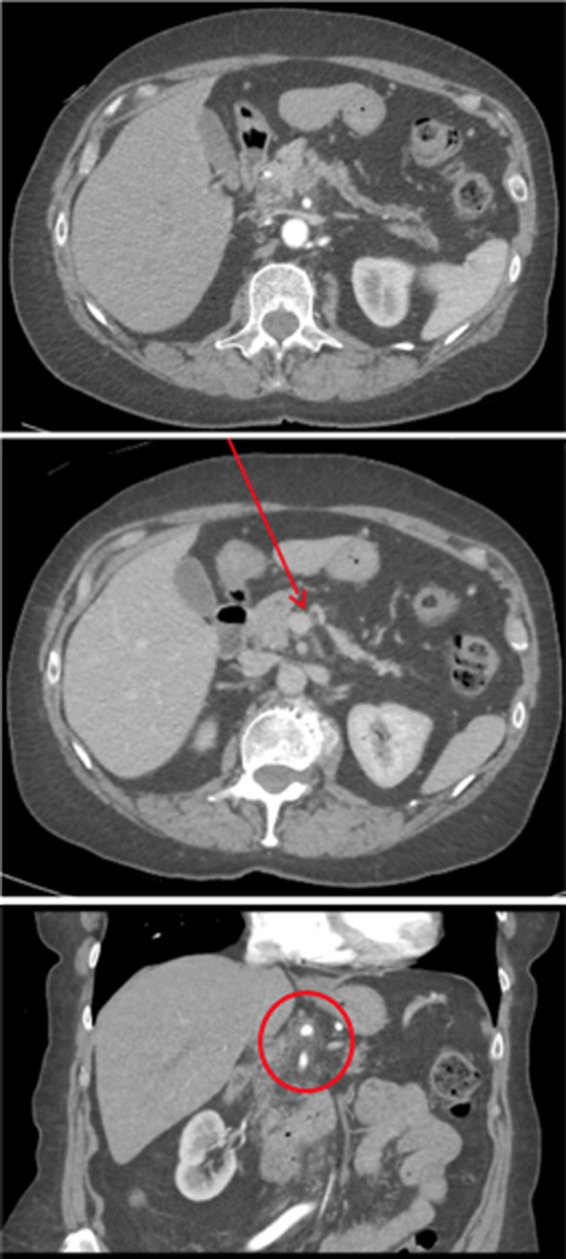
CT scan post multidrug neoadjuvant chemotherapy with a poorly defined infiltrative pancreatic body mass measuring ∼25 × 15 mm (top) with ill-defined soft tissue encasing the proximal splenic artery, common hepatic artery, distal celiac axis, and superior mesenteric artery (bottom). The mass (arrow) also abuts the portal vein (middle) and superior mesenteric vein.

A 74-year-old female patient presented with a locally advanced tumor of the proximal pancreatic body. The patient showed no signs of obstructive jaundice and did not require biliary stenting. Initially, her tumor was noted to involve the major visceral vessels, including the celiac artery, portal vein, and splenic artery and vein (Fig. 1). The tumor was deemed unresectable and the patient underwent extensive chemotherapy with multiple cycles of gemcitabine, Abraxane, 5-fluorouracil, Alloxantin, Avastin, and Xeloda. After showing a favorable response, she was referred for surgical re-evaluation. The post neoadjuvant chemotherapy CT scan (Fig. 2) showed a poorly defined infiltrative pancreatic neck and body mass measuring ∼25 × 15 mm with ill-defined soft tissue encasing the proximal splenic artery, common hepatic artery (CHA), distal celiac axis, and superior mesenteric artery (SMA). The mass also partially encased the portal vein and superior mesenteric vein (SMV). There was no radiologic evidence of hepatic metastasis and there was mild pancreatic duct dilatation. Pre-treatment and post-treatment serum CA 19–9 levels were 46 and 9, respectively. The patient did not receive any additional studies to assess GDA flow or the need for preoperative coiling/embolization. The absolute need for resection of the celiac axis was not determined until the time of the operation. The patient's functional status, perioperative risk, and likelihood of response were weighed and she was determined to be a candidate for an attempt at a modified Appleby procedure. After being properly informed of her various treatment options, she elected to undergo the operation.

Exploration of the duodenum and pancreas revealed a firm mass in the body of the pancreas with a soft pancreatic neck. No gross evidence of metastatic disease was present. There was a benign appearing lesion in the superior aspect of liver segment II, which was excised in its entirety and confirmed to be benign. Cholecystectomy was performed, followed by Kocherization of the duodenum and dissection of the pancreas and major vessels. We were fastidious in preserving the GDA. The splenic artery was controlled distal to the tumor, leaving a normal CHA pulse. The spleen and pancreatic body and tail were then serially elevated out of the retroperitoneum. The aorta was exposed, taking down the diaphragmatic crura. The celiac artery was identified at its origin, tied, divided, and oversewn with 5–0 polypropylene suture. Abnormal soft tissue was palpated along the proximal CHA. In light of this vessel involvement, we divided the distal CHA near the GDA. We then dissected the specimen off the SMA successfully and divided the pancreatic neck.

The inferior mesenteric vein was then clamped and tied. The inflamed pancreatic neck and proximal body were then dissected free from the right lateral aspect of the SMV and portal vein. The splenic vein was taken flush with the SMV, and its stump was oversewn, leaving good forward flow of the SMV to the portal vein. The proper hepatic artery (PHA) and the GDA were preserved throughout the resection. A Doppler ultrasound probe was used to test the GDA and PHA. Both arteries had adequate signal and in fact, the PHA had a faint palpable pulse. The liver parenchyma was also found to have a strong arterial signal. The specimen had two short stitches and purple dye placed at the neck margin and the rest of the specimen was inked per our Jefferson protocol.

Pathology of the resected specimen showed ductal adenocarcinoma with marked treatment effects with invasion of tumor into the peripancreatic soft tissue. The excision margins were free of neoplasia and the specimen had no regional lymph node metastasis (0/26). The tumor was within 2.0 cm from the pancreatic resection margin and within 0.1 cm of the circumferential margin in the posterior peripancreatic tissue. The maximum diameter of the tumor was 3.0 cm in size.

In the initial postoperative period, the patient had a transient transaminitis. A postoperative hepatic vascular ultrasound showed good hepatic arterial flow through the PHA and the transaminases normalized. The patient was discharged on postoperative day 9. She remains well without evidence of disease 7 months postoperatively, and has elected to receive no further chemotherapy.

## Discussion

Surgical resection of pancreatic cancer continues to be the mainstay of treatment. With advances in neoadjuvant therapy, surgical technique, and post surgical care, the definition of resectability has recently broadened to include those with celiac and hepatic arterial involvement. As evidenced by this case, patients with CHA involvement may become resectable after neoadjuvant therapy, and an aggressive surgical approach such as the modified Appleby procedure may offer margin negative resection.

Okada et al. showed that survival of patients with more advanced cancer who underwent a modified Appleby procedure was similar to patients with less advanced cancer undergoing a standard DP. The study also showed that patients with R0 resection had better survival than those with R1 or R2 resection, regardless of operation. A significant percentage of patients who underwent a DP for tumors within 10 mm of the splenic artery origin were found to have positive microscopic margins.^9^ In consideration of these positive margins, they recommended that patients with tumor located within 10 mm of the origin of the splenic artery undergo a modified Appleby operation. Sperti et al. demonstrated similar findings and showed that the DP-CAR increased the chances for R0 resection without increasing operative mortality.^10^

Bockhorn et al. looked at en bloc arterial resection (referring to celiac artery, CHA, or SMA resection) for pancreatic masses. The retrospective study showed no significant difference in median survival time in patients who underwent en bloc arterial resection compared with patients not requiring vascular resection. However, there was a significant increase in median survival of those undergoing resection compared to patients who underwent only palliative bypass.^11^

Takahashi et al. on the other hand showed a significantly shorter mean survival time in patients undergoing DP-CAR when compared to standard DP.^12^ There was, however, a trend toward more DP-CAR patients receiving R1 resection (44% vs. 22% *p* = 0.127) when compared to DP alone, which may have skewed the survival data of this report. Latona et al. recently reported excellent results in a small series of patients undergoing arterial reconstruction with the modified Appleby procedure.^13^

Patients who are deemed to have unresectable pancreatic cancer typically undergo nonsurgical treatment. Patients who fall into this category undergo chemotherapy followed by radiation. Interestingly, some of these patients undergo post-treatment imaging that demonstrates regression of disease, which make surgical resection feasible. Faris et al. looked at the conversion to resectability in patients with locally advanced pancreatic cancer after receiving neoadjuvant therapy with FOLFIRINOX.^6^ Five of their 22 patients identified were able to undergo R0 resections following therapy. Ferrone et a. showed improved traditional pathologic predictors of survival in patients who underwent similar therapy^7^ and Christians et al. demonstrated favorable resection rates following neoadjuvant therapy with FOLFIRINOX and chemoradiation.^8^

The assessment of true arterial involvement in these patients is very difficult preoperatively. In fact, many patients who receive DP-CAR subsequently are found to have tumors without intramural arterial invasion. Despite that fact, the DP-CAR also provides an R0 resection in a high percentage of patients. The outcome of interest would be if DP-CAR results in better local control and lower recurrence rate. This is not currently known and will likely never be tested by a randomized controlled trial. Strasberg and Fields site a strategy similar to our patient.^14^ She received neoadjuvant therapy in hopes of downsizing her tumor. The tumor was able to be downsized, but there was still need for arterial resection based on intraoperative findings of firm tissue encasing the celiac axis, which could not be differentiated between residual tumor or post-therapy fibrosis. The tumor did not approach the celiac axis at final pathology, though the nearest margin was 1 mm into the posterior peripancreatic tissue.

## Conclusion

Pancreatic cancer is a disease with high mortality and surgical resection remains the only definitive treatment. An R0 resection has been shown to significantly increase the survival of patients with pancreatic cancer. A pancreatic body or tail mass encasing the celiac vessels should not be an immediate referral for palliative care. Patients treated with neoadjuvant therapy and subsequent restaging that shows stable disease or regression may be offered an attempt at resection. When neoadjuvant therapy is followed by resection at a high-volume institution a favorable outcome can be produced. Therefore, in the appropriate patient population, en bloc vascular resection with an Appleby procedure can be done with acceptable perioperative morbidity and mortality when coupled with neoadjuvant therapy.

## Abbreviations Used

CHA: common hepatic artery
CT: computed tomography
DP: distal pancreatectomy
DP-CAR: DP-celiac axis resection
GDA: gastroduodenal artery
PHA: proper hepatic artery
SMA: superior mesenteric artery
SMV: superior mesenteric vein
